# Benchmarking and integration of methods for deconvoluting spatial transcriptomic data

**DOI:** 10.1093/bioinformatics/btac805

**Published:** 2022-12-14

**Authors:** Lulu Yan, Xiaoqiang Sun

**Affiliations:** School of Mathematics, Sun Yat-sen University, Guangzhou 510275, China; School of Mathematics, Sun Yat-sen University, Guangzhou 510275, China

## Abstract

**Motivation:**

The rapid development of spatial transcriptomics (ST) approaches has provided new insights into understanding tissue architecture and function. However, the gene expressions measured at a spot may contain contributions from multiple cells due to the low-resolution of current ST technologies. Although many computational methods have been developed to disentangle discrete cell types from spatial mixtures, the community lacks a thorough evaluation of the performance of those deconvolution methods.

**Results:**

Here, we present a comprehensive benchmarking of 14 deconvolution methods on four datasets. Furthermore, we investigate the robustness of different methods to sequencing depth, spot size and the choice of normalization. Moreover, we propose a new ensemble learning-based deconvolution method (EnDecon) by integrating multiple individual methods for more accurate deconvolution. The major new findings include: (i) cell2loction, RCTD and spatialDWLS are more accurate than other ST deconvolution methods, based on the evaluation of three metrics: RMSE, PCC and JSD; (ii) cell2location and spatialDWLS are more robust to the variation of sequencing depth than RCTD; (iii) the accuracy of the existing methods tends to decrease as the spot size becomes smaller; (iv) most deconvolution methods perform best when they normalize ST data using the method described in their original papers; and (v) the integrative method, EnDecon, could achieve more accurate ST deconvolution. Our study provides valuable information and guideline for practically applying ST deconvolution tools and developing new and more effective methods.

**Availability and implementation:**

The benchmarking pipeline is available at https://github.com/SunXQlab/ST-deconvoulution. An R package for EnDecon is available at https://github.com/SunXQlab/EnDecon.

**Supplementary information:**

Supplementary data are available at *Bioinformatics* online.

## 1 Introduction

The emergence of spatial transcriptomics (ST) has brought new opportunities for studying spatial heterogeneity of tissue architecture (Rao *et al.*, 2021) and cellular interaction (Larsson *et al.*, 2021). However, a major limitation of current ST technologies [e.g. Spatial Transcriptomics (Patrik *et al.*, 2016), 10× Visium (Genomics, 2019) and Slide-seq (Stickels *et al.*, 2021)] is that the measured gene expressions at one capture location (i.e. spot or grid) are from a mixture of multiple cells. This disadvantage hinders accurate quantification of spatial cellular distribution and downstream analysis.

To address the above issues of ST data, various computational methods have been developed to decompose spatial mixtures of each ST spot into individual cell types with the aid of single-cell RNA-seq (scRNA-seq) data (Longo *et al.*, 2021). For example, enrichment-based methods [e.g. Seurat (Stuart *et al.*, 2019) and MIA (Moncada *et al.*, 2020)] calculate the importance score or probability of the presence of different cell types in each spot. While other deconvolution methods aim to infer the proportion of cell types at each spatial location by employing linear regression models [e.g. SPOTlight (Elosua-Bayes *et al.*, 2021), spatialDWLS (Dong and Yuan, 2021)], probabilistic models [e.g. RCTD (Cable *et al.*, 2022), cell2location (Kleshchevnikov *et al.*, 2020)] or deep learning methods [e.g. DSTG (Song and Su, 2021)]. Additionally, a few reference-free methods [e.g. STdeconvolve (Miller *et al.*, 2022)] that deconvolve ST data without the aid of scRNA-seq data, have also been proposed.

Given the rapid development of these computational methods for ST deconvolution, it is important to quantitatively assess their performance and robustness for better applications. Recently, Li *et al.* (2022) evaluated the performance of several integration methods for predicting the spatial distribution of undetected transcripts and deconvoluting cell types. However, they only evaluated the impact of expression sparsity and normalization on the methods for predicting transcript distribution, but did not assess the impact of these factors on cell type deconvolution methods. Moreover, their study did not include all available state-of-the-art deconvolution methods for benchmarking. Therefore, a thorough evaluation of those deconvolution methods is still lacking.

Here, we present a comprehensive evaluation of the performance of 14 deconvolution methods on four datasets, including three synthetic ST datasets with known single-cell compositions and a human heart ST dataset. We quantitatively evaluate the accuracy of these methods by calculating the root-mean-square error (RMSE), Pearson correlation coefficient (PCC) and Jensen-Shannon divergence (JSD) between the predicted cell type compositions and the known compositions. Furthermore, we assess the stability of these deconvolution methods to the variation in sequencing depth, spot size and normalization choice. We also compare the computational resources consumed by different deconvolution methods. Moreover, we propose an ensemble learning-based deconvolution method, EnDecon, by aggregating different methods for more accurate deconvolution of ST data.

## 2 Materials and methods

### 2.1 Overview of ST deconvolution methods

The existing ST deconvolution methods are (in alphabetical order) cell2location, DestVI, DSTG, Giotto/Hypergeometric, Giotto/PAGE, Giotto/rank, MIA, RCTD, Seurat, spatialDecon, spatialDWLS, SPOTlight, STdeconvolve, stereoscope, STRIDE and Tangram. These methods can be mainly divided into four categories: enrichment scoring method, regression model-based deconvolution, probabilistic model-based deconvolution and deep learning model-based deconvolution. Enrichment-based methods [e.g. Seurat (Stuart *et al.*, 2019), Giotto-PAGE/rank/Hypergeometirc (Dries *et al.*, 2021; Kim and Volsky, 2005) and MIA (Moncada *et al.*, 2020)] usually infer the probability of the presence of each cell type in the spot based on an enrichment score of a gene set (e.g. cell-type-specific marker genes identified from scRNA-seq data). The other three categories of methods directly infer the proportions of different cell types within each spot. Toward that, regression model-based deconvolution methods [e.g. SPOTlight (Elosua-Bayes *et al.*, 2021), spatialDWLS (Dong and Yuan, 2021; Tsoucas *et al.*, 2019) and spatialDecon (Danaher *et al.*, 2022)] assume that a spot profile is a linear combination of cell-type-specific expression profile and cell type proportions. Alternatively, probabilistic model-based deconvolution methods [e.g. RCTD (Cable *et al.*, 2022), cell2location (Kleshchevnikov *et al.*, 2020), stereoscope (Andersson *et al.*, 2020), DestVI (Lopez *et al.*, 2022) and STdeconvolve (Miller *et al.*, 2022)] are to fit a probability distribution based on a statistical model, which assumes that the spatial gene expression follows a distribution, such as the Poisson distribution (Cable *et al.*, 2022) or negative binomial distribution (Andersson *et al.*, 2020). In addition, deep learning model-based methods [e.g. DSTG (Song and Su, 2021) and Tangram (Biancalani *et al.*, 2021)] deconvolute ST spots by borrowing information from scRNA-seq data. The principles and characteristics of these methods are described in Supplementary Text S1.

Notably, MIA does not release its code, and DSTG cannot output cell type information for comparison with the ground truth, so we benchmark the other 14 methods in this study.

### 2.2 Dataset collection and preprocessing

*Mouse embryo ST data*. It is a single-cell resolution ST data generated by sci-Space technology (Sanjay R. Srivatsan *et al.*, 2021), including 14 mouse embryo sections at different developmental stages. In this study, we selected the ST data coming from the 14th completely developed mouse embryo section for benchmarking, which contains 18 cell types and 17 301 cells with 52 535 genes per cell.

*MPOA ST data*. It is a single-cell resolution spatial expression dataset generated by applying the multiplex error-robust fluorescence in situ hybridization (MERFISH) technology to the mouse medial preoptic area (MPOA) (Moffitt *et al.*, 2018). We referred to the steps in STdeconvolve (Miller *et al.*, 2022) for the processing procedure of this dataset. The processed data consists of 9 cell types and 59 651 cells with 135 genes per cell.

*Mouse brain scRNA-seq and ST data*. The scRNA-seq data were sequenced by Smart-seq2 technology (Tasic *et al.*, 2016), including 4785 cells with 34 617 genes per cell. All cells were annotated into 15 clusters. The ST data of the mouse brain were obtained from 10X Genomics (2019). In this study, we selected the frontal cortex region for benchmarking, which consists of 1075 spots with each spot containing 31 053 genes.

*Human developing heart scRNA-seq and ST data*. The ST data of the human heart at three developmental stages [4.5–6, 6.5 and 9 post-conception weeks (PCW)] was obtained using Spatial Transcriptomics technology (Asp *et al.*, 2019). In this study, we selected the ST data at 6.5 PCW for deconvolution, which contains 1515 spots with 38 855 genes per spot. A set of scRNA-seq data generated in the same study was used as a reference for ST cell type annotation. The scRNA-seq data contains 15 clusters and 3777 cells in total, with 10 538 genes per cell.

We summarized the information of the above datasets in Table 1. The following steps were performed for pre-processing these datasets: (i) removing genes (rows) with row sum 0; (ii) filtering genes expressed in less than 5% of the cells or spots; (iii) retaining the cell types with at least 25 cells. Of note, since the MPOA ST dataset only has 135 genes, the step (i) was not performed for this dataset.

**Table 1.** The information of the collected datasets

Tissue	Technology	Resolution	Spot/cell number	Gene number
Mouse embryo	sci-Space	Single cell	17 301	52 535
MPOA	MERFISH	Single cell	59 651	135
Mouse brain	Smart-seq2	Single cell	4785	34 617
	10X Genomics	Spot	1075	31 053
Human	GemCode	Single cell	3777	10 538
developing heart	ST	Spot	1515	38 855

### 2.3 Generation of synthetic ST datasets for benchmarking

To benchmark different deconvolution methods, we employed different strategies for single-cell ST data and scRNA-seq data to synthesize multi-cells-per-spot datasets with known cell type compositions.

The mouse embryo ST dataset sequenced by sci-Space technology is at single-cell resolution for gene expression. However, the sci-Space technology uses spatially gridded barcodes to sequence tissue, so multiple cells within the same space grid are labeled with the same coordinate. Therefore, we treated a space grid as a simulated spot and aggregated the expression profiles of all cells in the grid to represent the expression profile of a simulated spot. The final synthetic ST dataset included 1393 spots with 52 535 genes per spot. We used the resulting cell type propositions of each spot as the ground truth.

For the MPOA ST data, we defined a square with a size 100×100 (∼100 µm in diameter), which was viewed as a spot-like region. The transcriptome profile of each simulated spot was simulated as the sum of expression profiles of all cells located in the region, and the coordinate of the simulated spot was set as the location of the starting cell (i.e. the upper left cell) in a square region. The final simulated spatial dataset contained 3072 regular spots with 135 genes per spot. The percentage of cell types in each spot was calculated as the ground truth.

For the mouse brain tissue, we first mapped the scRNA-seq dataset to the spatial locations of the paired ST dataset using the CellTrek tool (Wei *et al.*, 2022), resulting in a simulated single-cell resolution ST data. The synthetic spatial data had not only the scRNA-seq-like gene expression but also spatial location information. We then defined a square with a size of 150×150 (∼100 µm in diameter) and treated it as a simulated spot. The gene expressions of multiple cells in a square were aggregated to represent the spot-level expression profiles, and the location of the starting cell in the square was defined as the coordinate of the spot. Finally, the synthetic ST dataset contained 739 spots in total and each spot had 34 617 genes. We used the original cell-type label of each cell to calculate the percentage of cell types in each spot and viewed it as the ground truth.

The resulting three synthetic ST datasets are referred to as the embryo (sci-Space) dataset, MPOA (MERFISH) dataset and mouse brain (mapped sc-ST) dataset, respectively, in the following text, and Table 2 summarizes important statistics of these three datasets.

**Table 2.** The information of the synthetic datasets

Dataset	Spot number	Gene number	Cluster number
embryo (sci-Space)	1393	52 535	18
MPOA (MERFISH)	3072	135	9
mouse brain (mapped sc-ST)	739	34 617	15

Furthermore, to investigate the impact of different sequencing depths, spot sizes and data normalization choices on the performance of deconvolution methods, we synthesize datasets with different sequencing depths or different spot sizes and processed the above three ST datasets using different data normalization methods. See details in Supplementary Texts S2–S4.

### 2.4 Implementation of deconvolution methods

We followed the instructions provided on the website of each tool to implement deconvolution. The details of the implementation of the existing 14 deconvolution methods are described in Supplementary Text S5.

### 2.5 Evaluation metrics

We used RMSE, PCC and JSD (see details in Supplementary Text S6) to evaluate the performance of different deconvolution methods by using the known cell type proportions in the three synthetic ST datasets as the ground truth.

### 2.6 Integrating deconvolution methods

After benchmarking different deconvolution methods, we developed an ensemble learning-based deconvolution method (EnDecon) for ST data by drawing on strengths from existing methods. Notably, to ensure the efficiency of EnDecon, we integrated three top-performing methods into a linear weighted model as follows,

(1) $$y=w_{1}x_{1}+w_{2}x_{2}+w_{3}x_{3}$$

where $x_{1}$, $x_{2}$ and $x_{3}$ represent the cell type proportions derived from the top three individual methods, respectively, and $w_{1}$, $w_{2}$ and $w_{3}$ are the corresponding weights.

The weights in the above model were trained using 10-fold cross-validation method. Specifically, we divided the embryo (sci-Space) dataset and mouse brain (mapped sc-ST) dataset into 10-folds, respectively. For each dataset, we took turns choosing 9-folds out of 10-folds as the training set to estimate the coefficients (i.e. $w_{1}$, $w_{2}$ and $w_{3}$) and using the remaining 1-fold of the two datasets as the testing sets for calculating RMSE, PCC and JSD. We conducted the above 10-fold cross-validation 10 times and took the average of the estimated values of each coefficient as the final weight for each of the three individual methods in the EnDecon. Furthermore, we validated the performance of the trained EnDecon model on the independent MOPA (MERFISH) dataset.

## 3 Results

### 3.1 Benchmarking framework

**Fig. 1.**
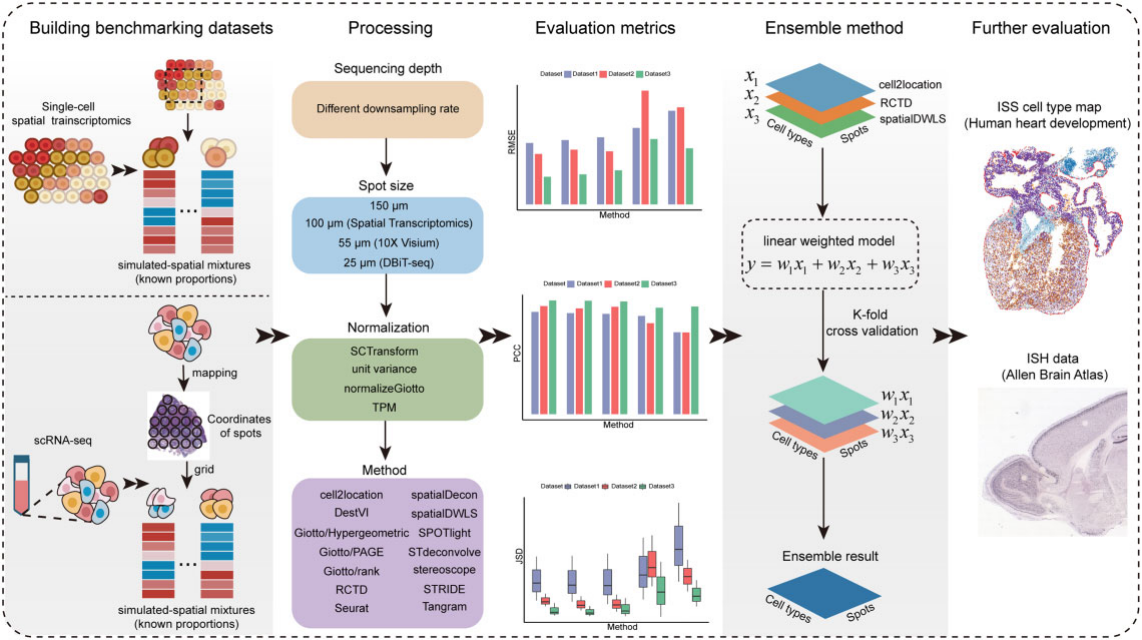
Schematic diagram of the benchmarking workflow to compare the performance of the 14 methods for deconvoluting ST data. Firstly, we adopted two strategies to build benchmark datasets based on single-cell resolution ST and scRNA-seq datasets. Next, we evaluated the prediction accuracy of the 14 deconvolution methods using three metrics (RMSE, PCC and JSD) and assessed the impact of different factors (sequencing depth, spot size and ST normalization choice) on deconvolution results. Furthermore, we developed an ensemble method by weighting and integrating the top 3 individual methods. Lastly, we adopted ISS data and ISH data to further evaluate the performance of all methods

To test the performance of 14 deconvolution methods, we designed a benchmarking workflow as shown in Figure 1. Briefly, we first collected both single-cell resolution ST data and scRNA-seq data to synthesize the low-resolution ST datasets with known cell type compositions (see details in Section 2.3). We then assessed the accuracy of each method by calculating RMSE, PCC and JSD between the predicted cell type compositions and the ground truth based on the above synthetic ST datasets. We next examined the impact of sequencing depth, spot size and normalization choice on the deconvolution results and assessed the time and space complexities of different methods. Furthermore, we developed an ensemble model by integrating the top three deconvolution methods ranked according to the benchmarking results with a linear weighted model. We adopted the *k*-fold cross-validation method to train the weights in the ensemble model and further tested and compared its performance using independent validation dataset.

### 3.2 Performance evaluation of 14 deconvolution methods

**Fig. 2.**
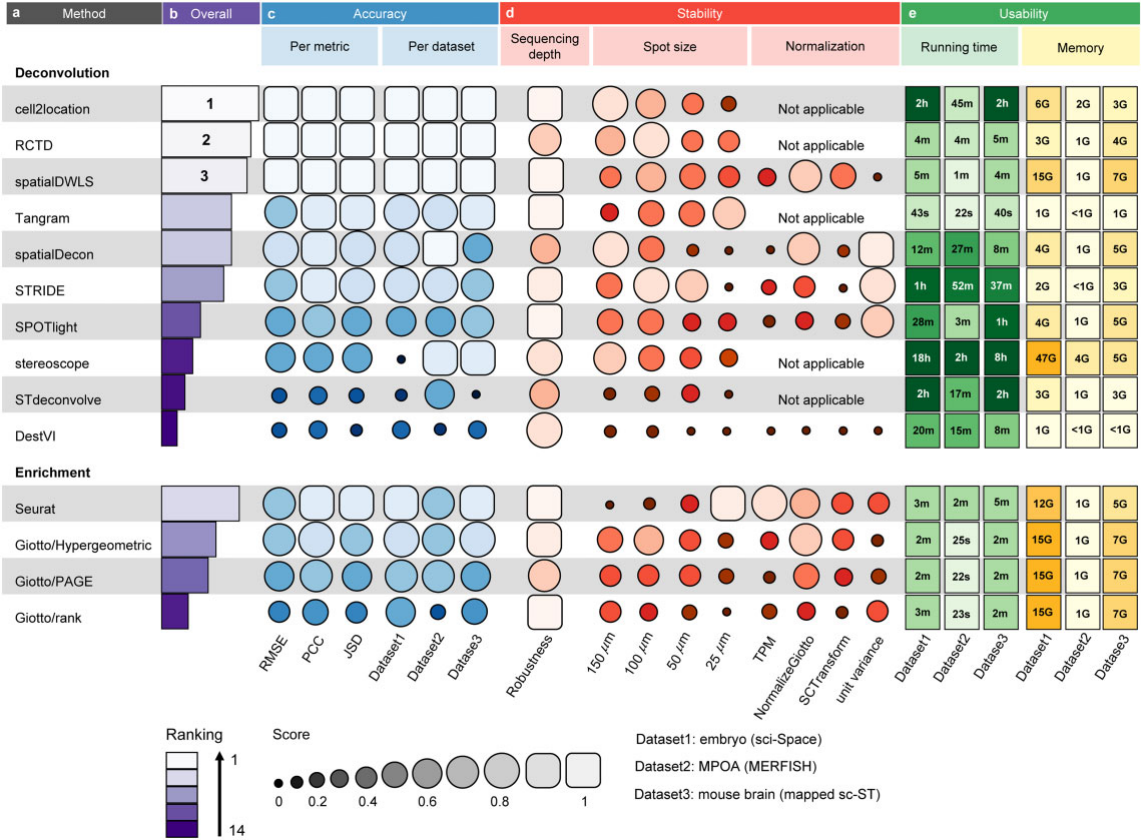
Summary of benchmarking results for the 14 deconvolution methods. (**a**) The names of two types of methods (i.e. enrichment-based methods and deconvolution-based methods) are respectively ordered by their performances. (**b**) The overall ranking of the 14 methods. (**c**) Accuracy of different methods across three metrics and three synthetic datasets. (**d**) Stability of each deconvolution method with respect to sequencing depth, spot size and normalization. To evaluate the robustness of different methods to sequencing depth, for each method, we calculated the variance of the aggregated score at different sequencing depths (Supplementary Text S6). ‘Not applicable’ indicates that certain deconvolution method does not support performing normalization of ST data. (**e**) Usability assessment in terms of running time and memory

Based on the three synthetic datasets, we evaluated the performance of each method in the following three aspects: (i) prediction accuracy in terms of cell type proportion deconvolution, evaluated using the metrics including RMSE, PCC and JSD; (ii) stability of the method with respect to the sequencing depth, spot size and normalization choice of the ST data; (iii) usability of the tool in terms of running time and memory. Overall, we found that cell2location has the best performance, followed by RCTD and spatialDWLS (Fig. 2a and b). Below we described the benchmarking results in more detail.

**Accuracy.** We compared the accuracy of different deconvolution methods across metrics and datasets. We found that the accuracy of each of the 14 methods was generally quite stable across the three metrics, but the accuracy of some methods (i.e. stereoscope, STdeconvolve and Giotto/rank) varied depending on datasets (Fig. 2c).

Specifically, we compared the values of RMSE, PCC and JSD of 14 methods across the three synthetic datasets (Supplementary Fig. S1). We found that cell2location, RCTD and spatialDWLS outperformed the other methods. For instance, on the mouse brain (mapped sc-ST) dataset, the RMSE values of cell2location (0.0373), RCTD (0.0407) and spatialDWLS (0.0461) were lower than those of other methods (Supplementary Fig. S1a). Consistently, the PCC values of cell2location (0.9837), RCTD (0.9807) and spatialDWLS (0.9748) were higher than those of other methods (Supplementary Fig. S2b). Moreover, the median JSD values of cell2location, RCTD and spatialDWLS were 0.0143, 0.0120 and 0.0188, respectively, which were lower than the median JSD values of other methods (Supplementary Fig. S2c). In addition, comparing RMSE or PCC per cell type on the three synthetic ST datasets (Supplementary Fig. S2) consistently demonstrated that cell2location, RCTD and spatialDWLS have smaller RMSE or PCC values for individual cell types.

To evaluate the performance of the deconvolution methods more intuitively, we reconstructed the spatial cell type distribution maps for the synthetic ST datasets of the mouse embryo, MPOA (one certain section) and mouse brain tissues according to the deconvolution proportions and compared them with the gold standard. For the embryo (sci-Space) dataset, cell2location, RCTD and spatialDWLS well reconstructed the layered structure and accurately deconvoluted ST spots (Supplementary Fig. S3). Specifically, these three methods correctly mapped the main cell type in the cortex region, neuron, to the top and right limbic layers, and mapped the Erythroid Lineage and Hepatocytes cell types mainly to the middle layer, i.e. the liver area in the mouse embryo (Supplementary Fig. S3a). The spatial patterns of these cell types were consistent with those in the gold standard (Supplementary Fig. S3b). In contrast, the cell types predicted by SPOTlight in the overall embryo region were mostly Schwann cells or Radial glia cells, which was not comparable to the gold standard. Besides, the spatial deconvolution of DestVI and Giotto/rank failed to reveal spatial heterogeneity of the tissue. For the MPOA (MERFISH) dataset, the spatial locations of the Ependymal cells predicted by cell2location, RCTD and spatialDWLS were in the middle of this section, highly consistent with those of the gold standard (Supplementary Fig. S4).

Furthermore, to assess the predicted spatial cell type distribution of all methods within the mouse brain cortex structure, we used the expression pattern of the known cell-type marker gene in the ISH image data from the Allen Mouse Brain Atlas as the gold standard. In this study, we used Rasgrf2, Plcxd2 and Cplx3 as the marker genes for three cortex cell types, L2/3, L4 and L6b, respectively, as reported by Zeisel *et al.* (Amit Zeisel *et al.*, 2015) (Supplementary Figs S5–S7). The proportions of these three cell types estimated by cell2location, RCTD and spatialDWLS were highly consistent with the corresponding marker gene expressions in the ISH images (Supplementary Figs S5–S7). Specifically, the L2/3 subcluster was mapped to the lateral border of the cortex at larger proportions, and L4–L6b subclusters were predicted to line up along the stretched area descending toward the center. The spatial organization of these cell types agreed with the strictly layered structure of the cortex. However, the spatial locations of L2/3 and L4 predicted by spatialDecon and SPOTlight were distributed throughout the cortex region.

**Stability.** To test the stability of each method, we investigated the impact of different sequencing depths, spot sizes and normalizations on the deconvolution results of each method (Fig. 2d).

Firstly, most methods were rather robust to the variation of sequencing depth, while RCTD, spatialDecon and stereoscope were more sensitive to changes in sequencing depth (Fig. 2d). Specifically, on the mouse brain (mapped sc-ST) dataset, RCTD and stereoscope only performed well when the sequencing depth was low, while spatialDecon became gradually worse when the sequencing depth decreased (Supplementary Fig. S8). In general, cell2location and spatialDWLS had the best performance under different sequencing depths.

Secondly, the performance of all methods except Tangram and Seurat tended to become worse when the spot size decreased from 150 to 25 µm (Fig. 2d). Of note, Seurat outperformed all the other methods on the MPOA (MERFISH) dataset at spot size = 25 µm, and was no longer the best at larger spot sizes. Furthermore, the performance of STdeconvolve was better than that of DestVI on the mouse brain (mapped sc-ST) dataset with spot size = 25 µm, whereas this observation no longer held when spot size became larger (Supplementary Fig. S9). Overall, cell2location, RCTD and spatialDWLS still maintained good performance when spot sizes varied.

Thirdly, the performance of deconvolution methods varied widely concerning different normalization methods, suggesting that there was no ‘one size fits all’ normalization approach that works for all deconvolution methods (Fig. 2b). Particularly, Giotto/Hypergeometric, Giotto/PAGE, Giotto/rank and spatialDWLS performed best on the embryo (sci-Space) and mouse brain (mapped sc-ST) datasets when employing the ‘normalizeGiotto’ method described in their respective original publications. SPOTlight, STRIDE and Tangram showed the best performance when using ‘unit variance’ to normalize the spatial gene expression matrix (Supplementary Fig. S10).

**Usability.** We tested the running time and memory usage of the 14 deconvolution methods on the same platform (2.7 GHz, 39 424 KB L3 Cache, 112 CPU cores).

Regarding running time, we observed that Tangram had the shortest running time on three datasets, and Giotto/Hypergeometric, Giotto/PAGE, Giotto/rank, RCTD, Seurat and spatialDWLS ran less than six minutes, while stereoscope ran more than two hours. Notably, for the three methods (i.e. cell2location, RCTD and spatialDWLS) with better performance, the running time of cell2location was nearly 100 times that of RCTD or spatialDWLS (Fig. 2e).

Regarding memory usage, we found that stereoscope had the highest RAM requirement, while Tangram had the lowest RAM requirement (Fig. 2e). Giotto/Hypergeometric, Giotto/PAGE, Giotto/rank and spatialDWLS had the same memory usage since the functions used by these four methods belong to the same package. Particularly, RCTD had the least memory usage among the top-ranked three methods (i.e. cell2location, RCTD and spatialDWLS).

In short, Tangram was the most efficient, while stereoscope was the least efficient. RCTD not only performed well among the 14 methods in terms of inferring cell type proportions for a given spot but also had higher efficiency.

### 3.3 Integration for an ensemble model

**Fig. 3.**
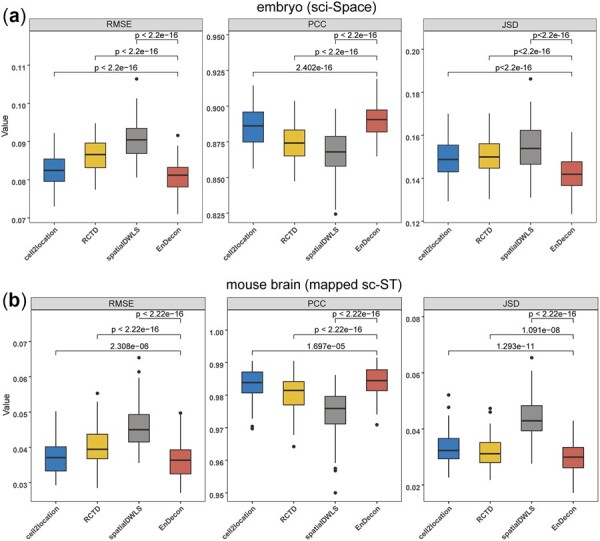
The performance of the EnDecon model on testing sets of the embryo (sci-Space) dataset (**a**) and mouse brain (mapped sc-ST) dataset (**b**). Ten times 10-fold cross-validation was performed. Accuracy metrics (i.e. RMSE, PCC and JSD) of EnDecon were compared to the other three individual methods (i.e. cell2location, RCTD and spatialDWLS). Wilcoxon rank sum test (one-tailed) *P*-value was calculated to assess the statistical significance of the difference

To test whether an ensemble of the existing methods could improve the accuracy of the ST deconvolution, we compared the performance of EnDecon with cell2location, RCTD and spatialDWLS on testing and validation sets. The cross-validation results (Fig. 3) showed that the ensemble model achieved significant performance improvement on testing sets of the embryo (sci-Space) and mouse brain (mapped sc-ST) datasets in terms of RMSE, PCC and JSD. Moreover, EnDecon outperformed the three individual methods on the independent validation set [i.e. MOPA (MERFISH) dataset] (Table 3) concerning all three metrics.

**Table 3.** Comparing the performance of the EnDecon model with each of the top three methods on the independent validation dataset

Dataset	Method	RMSE	PCC	JSD
MPOA	cell2location	0.0682	0.9346	0.0631
	RCTD	0.0740	0.9149	0.0562
(MERFISH)	spatialDWLS	0.0718	0.9289	0.0569
	**EnDecon**	**0.0544**	**0.9515**	**0.0484**

The boldface values indicate that the proposed method, EnDecon, outperformed other methods.

Furthermore, we depicted the spatial distributions on the three synthetic datasets using the cell type proportions predicted by EnDecon and compared them with the corresponding gold standard (Supplementary Fig. S11). We found a high degree of consistency between EnDecon’s predicted spatial distributions of cell type abundance and the gold standard on both the embryo (sci-Space) dataset (Supplementary Fig. S11a) and the MPOA (MERFISH) dataset (Supplementary Fig. S11b). For the mouse brain (mapped sc-ST) dataset, it was clear that the estimated cell type proportions by EnDecon agree with the corresponding marker gene expression in the ISH images (Supplementary Fig. S11c).

Finally, we compared the computational resources consumed by EnDecon and 14 other individual methods (Supplementary Fig. S12). We observed that the computational efficiency of EnDeon was comparable to that of cell2location and better than that of stereoscope. Taking the mouse brain (mapped sc-ST) dataset as an example, EnDecon ran a little bit slower than cell2location (i.e. nearly eight minutes slower) and EnDecon required less memory than spatialDWLS.

### 3.4 Application of different methods on real ST datasets

**Fig. 4.**
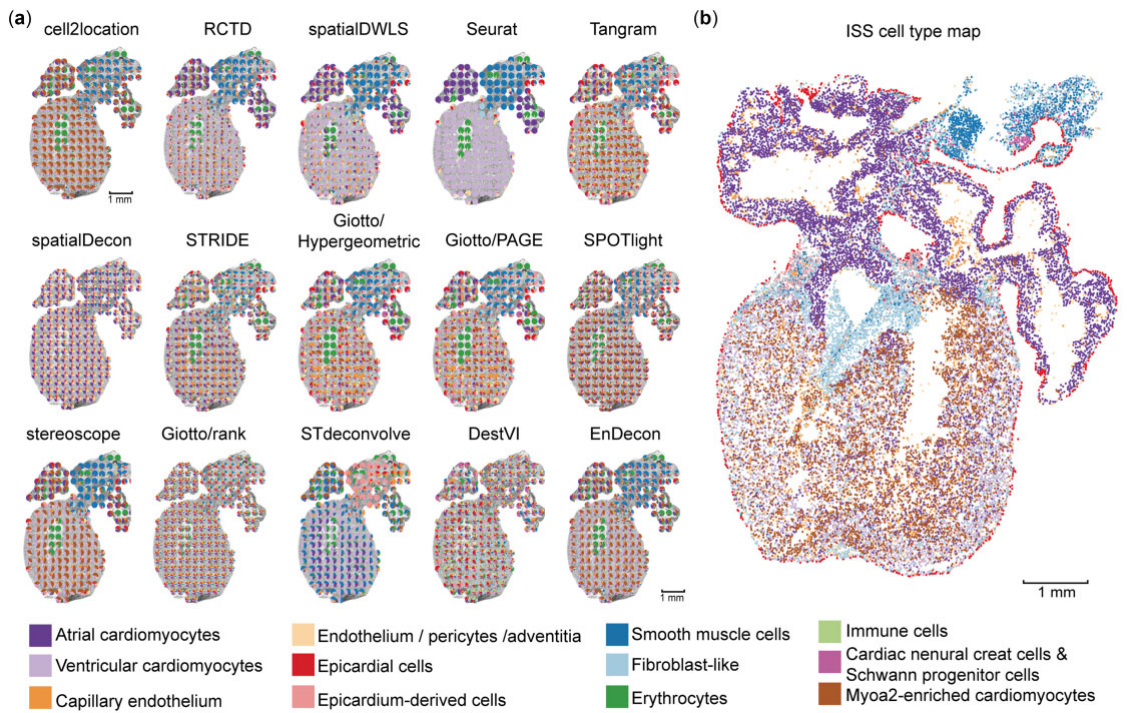
Application of different deconvolution methods on the human developing heart dataset. (**a**) The spatial distributions of cell type proportion predicted by 15 deconvolution methods in sample 4 at 6.5 PCW. Each pie represents the cell type proportions in each spot in the ST slide, and colors represent different cell types. (**b**) The spatial cell type map generated by integrating ISS and scRNA-seq data in the original study

To verify the performance of different deconvolution methods on real ST data, we applied them to study the spatial organization of the human developing heart ST dataset. A recent study provides a spatiotemporal atlas of the human developing heart (4.5–5, 6.5 and 9 PCW) by integrating scRNA-seq, ST and ISS data (Asp *et al.*, 2019). We performed cell type deconvolution on the ST data of samples at 6.5 PCW using 15 deconvolution methods (Fig. 4a) and used the ISS cell type map in the original study (Asp *et al.*, 2019) as the gold standard (Fig. 4b).

Using sample 4 at 6.5 PCW as an example, it is shown that the spatial locations of all cell types inferred by cell2location, RCTD, spatialDWLS, stereoscope and EnDecon well reconstructed the hierarchical structure of the human heart (Fig. 4a). Specifically, as expected, these methods mapped Ventricular and Atrial cardiomyocytes to the lower and upper ventricles. Also, Smooth muscle cells were correctly predicted to localize in the outflow tract, consistent with the spatial location of the corresponding cell types in the ISS cell type map (Fig. 4b). Of note, although Seurat estimated the clearest cell-type spatial structure, only a partial domain of cell types could be enriched. In fact, the ISS cell type map showed that the lower ventricle was mainly composed of Ventricular cardiomyocytes and Myoa2-enriched cardiomyocytes cell types, whereas Seurat inferred only the former in the lower ventricle. As such, the cell type deconvolution inferred by Seurat seems less favorable than that of cell2location, RCTD, spatialDWLS, EnDecon or even stereoscope.

## 4 Discussion

The emerging ST technologies provide new insights into spatial heterogeneity in cellular abundance and gene expression. However, the resolutions of most of the current ST data are not guaranteed to be single-cell. Therefore, it is necessary to quantify cell type abundance for individual spots in the ST data. Although many computational methods have been developed to address this challenge, their performances have not been comprehensively evaluated. In this study, we benchmarked 14 state-of-the-art methods for ST deconvolution in terms of accuracy, stability and usability. We further developed an ensemble model that significantly improved the deconvolution accuracy.

**Fig. 5.**
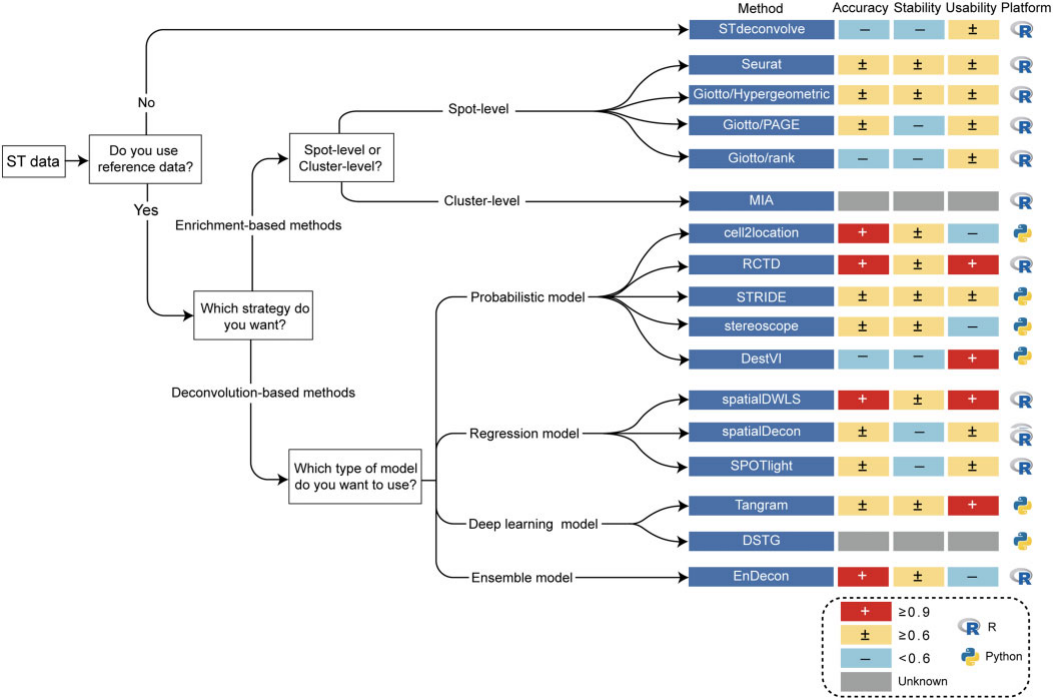
Practical guidelines for method users. As the performance of a method mainly depends on whether a reference is needed, the strategy is adopted and the mathematical model used, we, therefore, provide a set of practical guidelines combining the accuracy, stability and user-friendliness of the method. The methods on the right are ranked according to their performance on a specific (set of) deconvolution method. Further to the right, the accuracy, stability, usability scores (+: ≥0.9; ±: ≥0.6; –: <0.6) and operating platform are displayed in order. The grey square denotes that the corresponding method fails to evaluate

Based on the benchmarking results, we provide a practical guideline for researchers to choose suitable tools to analyze their ST datasets (Fig. 5). The performance of a deconvolution method heavily depends on whether it requires reference data, its deconvolution strategy (e.g. enrichment or deconvolution), and its modeling approach (e.g. linear regression model, probabilistic model). Therefore, we categorize them according to the above three factors, and for each category, our evaluation suggests an optimal choice, as shown in Figure 5.

Among all the 14 deconvolution methods, only STdeconvolve is reference-free while it performed not well. The other methods requiring scRNA-seq data as a reference can be categorized into enrichment-based methods and deconvolution-based methods. Among the enrichment-based methods, Seurat had the best performance, followed by Giotto/Hypergeometric, Giotto/PAGE and Giotto/rank. Of note, we did not evaluate MIA because the authors did not disclose executable code in the original publication (Moncada *et al.*, 2020). Particularly, the enrichment results of Seurat can be directly used as cell type proportions for subsequent analysis. In contrast, the enrichment scores estimated by the three methods included in the Giotto package (i.e. Hypergeometric, PAGE and rank) represent the importance of different cell types at each spot, which are required to be normalized to get ultimate cell type proportions.

Regarding the deconvolution-based methods, cell2location and RCTD performed best among all probabilistic model-based deconvolution methods. However, the usability of cell2location was worse than that of RCTD. Among regression model-based deconvolution methods, spatialDWLS was superior to spatialDecon and SPOTlight in terms of accuracy, stability and usability. Technically, spatialDWLS first uses enrichment analysis to identify the cell type at each spot and then applies a regression model to infer the proportion of the selected cell type, and these steps are performed on subclusters of ST data. This might explain why spatialDWLS outperforms other methods. Regarding deep learning model-based methods, Tangram had high efficiency and acceptable accuracy. Moreover, Tangram can predict the spatial distribution of undetected transcripts. Besides, DSTG was omitted for benchmarking since its deconvoluted result had no annotation information and could not be compared with the ground truth. Currently, only a few deep learning model-based deconvolution tools have been developed. We anticipate that deep learning methods have the potential to further improve the accuracy and stability of ST deconvolution with high computational efficiency.

To improve the deconvolution accuracy, we proposed an ensemble learning-based method to estimate cell type proportions for ST spots. By integrating the top three deconvolution methods using a linear weighted model, EnDecon achieved a significant improvement in deconvolution accuracy. Notably, EnDecon assigns a larger weight to a better method and meanwhile combines strengths from the three individual methods. Thus, EnDecon can naturally maintain a good performance under different settings (e.g. sequencing depth, spot size and normalization) and thus has better stability than the other individual methods. Therefore, EnDecon provides an alternative and more effective method for ST deconvolution.

In future studies, new methods could be developed for ST deconvolution by considering the constraint of adjacent spots. More specifically, for the ST data, we can consider synthesizing the adjacent spots into new spots containing more cells, which satisfies the linear constraint that the proportion of cell types in the simulated big spot is equal to the weighted sum of the proportion of cell types in the respective small spots. This constraint can increase the number of samples for deconvolution inference and achieve self-supervised learning. Currently, existing deconvolution methods do not consider this constraint, which may explain the observation that smaller spot sizes tend to have larger RMSE values in our study. New deconvolution methods incorporating this constraint may address the issue and are anticipated to improve deconvolution performance.

Another limitation of the current existing ST deconvolution methods is that most of them only infer cell type proportions but do not estimate cell-type-specific (CTS) gene expression at each spot, which is equally important for ST data analysis. For bulk RNA-seq data, many methods have been developed for CTS genes expression inference, such as TCA (Rahmani *et al.*, 2019), CIBERSORTx (Newman *et al.*, 2019), bMIND (Wang *et al.*, 2021) and swCAM (Chen *et al.*, 2022). However, for the ST data, only RCTD can infer the CTS gene expression at each spot. RCTD calculates CTS gene expression under the assumption that random effects of gene expression are shared across all cell types, which may lead to inaccurate estimation. Therefore, new methods are necessary and valuable to be developed for inferring CTS gene expression from ST data in the future.

In conclusion, this study performs a comprehensive comparison of available ST deconvolution methods for decomposing the cell type composition of spatial mixtures. The major new findings in this study are as follows: (i) cell2loction, RCTD and spatialDWLS are more accurate than other ST deconvolution methods, based on the evaluation of RMSE, PCC and JSD; (ii) cell2location and spatialDWLS are more robust to the variation of sequencing depth than RCTD; (iii) the accuracy of the existing methods tends to decrease as the spot size becomes smaller; (iv) most deconvolution methods only perform well when they normalize ST data using the method described in their original publications; and (v) the ensemble learning-based deconvolution method, EnDecon, achieves more accurate deconvolution of the ST data. The results provide valuable information and guideline for analyzing spatial transcriptome data and developing new deconvolution methods.

## Supplementary Material

btac805_Supplementary_Data

## Data Availability

The data used in this study are publicly available. The embryo (sci-Space) dataset was downloaded from the Gene Expression Omnibus database under GSE166692; the MPOA (MERFISH) dataset was downloaded from https://datadryad.org/stash/dataset/doi:10.5061/dryad.8t8s248/; the mouse brain ST data were download from https://www.dropbox.com/s/azjysbt7lbpmbew/brain_st_cortex.rds?dl=0 and the paired scRNA-seq data were downloaded from https://www.dropbox.com/s/ruseq3necn176c7/brain_sc.rds?dl=0; the human developing heart ST and scRNA-seq data were downloaded from https://data.mendeley.com/datasets/mbvhhf8m62/2. For further validation, the ISS data were obtained from https://doi.org/10.6084/m9.figshare.10058048.v1 and the ISH image data were downloaded from https://mouse.brain-map.org/.
