# Supplementary material for: Benchmarking and integration of methods for deconvoluting spatial transcriptomic data
Source: Bioinformatics. 2022 Dec 14;39(1):btac805. doi: 10.1093/bioinformatics/btac805 (PMC9825747; doi:10.1093/bioinformatics/btac805)
Supplement: btac805_Supplementary_Data [file btac805_supplementary_data.pdf]

Supplementary Information

Benchmarking and integration of methods for deconvoluting spatial transcriptomic data

Lulu Yan[1] and Xiaoqiang Sun[1,*]

[1] School of Mathematics, Sun Yat-sen University, Guangzhou 510275, China.

*To whom correspondence should be addressed.

Email: sunxq6@mail.sysu.edu.cn

Text S1 Principles and characteristics of ST deconvolution methods

The existing computational methods can be categorized into four categories: enrichment-based methods, regression model-based deconvolution methods, probabilistic model-based deconvolution methods, and deep learning model-based methods. The first category of methods aims to identify cell types contained in each spot by calculating enrichment scores representing the degree of match between gene signatures of those cell types and the expression pattern of the spot, while the latter three seek to infer the proportions of cell types for a given spot by using regression models, probabilistic models, or deep learning models, respectively. Below we overview the principles and characteristics of these methods.

Enrichment-based methods

- ***Seurat***. It embeds the ST data and scRNA-seq reference data into a shared low-dimensional space and identifies the pairwise correspondences between cells and spots (Stuart, et al., 2019). It computes the enrichment scores, representing the probability of the presence of each cell type in the spot, via the 'FindTransferAnchors' and 'LableTransfer' functions.

- ***Giotto***. The Giotto package (Dries, et al., 2021) includes three independent methods for calculating enrichment scores: 1) PAGE (Kim and Volsky, 2005) scores the fold change of cell-type-specific genes for each spot relative to other spots; 2) rank calculates enrichment scores based on the ranking of cell-type-specific genes from scRNA-seq data and the ranking of location-specific genes from ST data; 3) Hypergeometric test method evaluates the degree of enrichment of cell types at each spot by calculating a p-value.

- ***MIA***. Multimodal Intersection Analysis (MIA) (Moncada, et al., 2020) estimates an enrichment score of each cell type in a given spatial location by measuring the extent of overlap between cell-type-specific marker genes from scRNA-seq and spot-position-specific genes from ST data.

Regression model-based deconvolution methods

- ***SPOTlight***. It uses seed non-negative matrix factorization (sNMF) to obtain gene distributions from scRNA-seq data and subsequently adopts non-negative least squares (NNLS) to deconvolute spatial data (Elosua-Bayes, et al., 2021).

- ***spatialDWLS***. It extends the dampened weighted least squares (DWLS) approach (Tsoucas, et al., 2019) to deconvolute ST data, which identifies cell types of each spot by using enrichment analysis and then employs DWLS to infer cell type proportions (Garcia-Alonso, et al., 2021).

- ***spatialDecon***. It uses log-normal regression, instead of the classic core least-squares regression, to deconvolve ST data, which could largely correct the skewness and inconsistent variance of spatial gene expression data (Danaher, et al., 2022).

Probabilistic model-based deconvolution methods

- ***RCTD***. It estimates cell type compositions for each spot by fitting a statistical model, assuming that gene counts follow a Poisson distribution while correcting for differences between ST and scRNA-seq techniques (Cable, et al., 2022).

- ***cell2location***. It estimates the cell type signature from scRNA-seq data using a model based on negative binomial regression and then uses this reference signature to estimate the abundance of each cell type in each spot (Kleshchevnikov, et al., 2020).
- ***stereoscope***. It utilizes a probabilistic model to decompose cell type mixtures in spatial data, assuming that both spatial and scRNA-seq data follow a negative binomial distribution (Andersson, et al., 2020).
- ***STRIDE***. This method leverages latent Dirichlet allocation (LDA), a generative probabilistic model, trained from scRNA-seq data to decompose spot expression into individual cell types (Sun, et al., 2022).
- ***DestVI***. It uses a probabilistic method constructed by variational inference and latent variable models to obtain the cell type decomposition at each spot (Lopez, et al., 2021).
- ***STdeconvolve***. This method is built on an LDA model to infer the proportional representation of cell types in each multi-cellular spot, without relying on external single-cell reference (Miller, et al., 2022). So, it is a reference-free deconvolution method for ST deconvolution, taking into account of limited availability of suitable single-cell data as a reference due to technical and other reasons.

Deep learning-based deconvolution methods

- ***DSTG***. It deconvolutes ST data by leveraging graph convolutional networks trained from pseudo-ST data, where pseudo-ST data is generated by randomly combining expression data of scRNA-seq cells (Song and Su, 2021).
- ***Tangram***. It learns a spatial alignment for scRNA-seq data by using a deep learning framework and non-convex optimization, which allows decomposing ST data by assigning cells from scRNA data to spatial locations (Biancalani, et al., 2021).

Text S2 Generation of datasets with different sequencing depths

To test the robustness of different deconvolution methods concerning sequencing depth on three synthetic ST datasets, we adopted the 'downsampleMatrix' function in the DropletUtils package (https://www.bioconductor.org/packages/release/bioc/html/DropletUtils.html) to down-sample the spatial expression matrix to generate datasets with different sequencing depths. Specifically, the relatively lower number of genes captured by MERFISH and the lower counts per gene sequenced by sci-Space resulted in low resolution of the gene expression in these two synthetic ST datasets. Therefore, we down-sampled these datasets by setting the down-sampling rate = 1, 0.8, 0.6, 0.2, where the down-sampling rate = 1 implies the raw counts. For the mouse brain (mapped sc-ST) dataset, the total counts per spot consisted of the counts of multiple scRNA-seq cells, we then set the down-sampling rate = 1, 0.5, 0.2, 0.05, 0.01. Notably, the sequencing depth of the simulated dataset generated by the down-sampling rate = 0.01 was comparable to that of the real ST data. Therefore, subsequent experiments on the mouse brain (mapped sc-ST) dataset were performed based on simulated data with the down-sampling rate = 0.01. Table S1 lists details of the counts corresponding to different down-sampling rates on the three simulated datasets.

Text S3 Generation of datasets with different spot sizes

To investigate the impact of different spot sizes on the performance of different deconvolution methods, we defined squares with different areas to adjust the spot size, i.e., the number of cells contained in a spot. Since the square size of the embryo (sci-Space) dataset was defined during sequencing and was not adjustable during the simulation process, we did not test the impact of different spot sizes on this dataset. For MPOA (MERFISH) and mouse brain (mapped sc-ST) datasets, we generated simulated datasets with three different spot sizes, 25 μm, 55 μm, and 150 μm in diameter, respectively. Specifically, the simulated spot with a diameter 25 μm mimics the DBiT-seq technology (Liu, et al., 2020) and the spot with a diameter 55 μm mimics the Spatial Transcriptomics technology. For the MPOA (MERFISH) dataset, the spot sizes with diameters 25 μm, 55 μm, and 150 μm correspond to the square sizes with 25×25, 55×55, and 150×150, respectively. For the mouse brain (mapped sc-ST) dataset, the spot sizes with diameter 25 μm, 55 μm, and 150 μm correspond to the square sizes with 75×75, 105×105, and 200×200, respectively.

Text S4 Data normalization choices

To evaluate the impact of different normalization methods on the performance of deconvolution methods, we processed ST data using four normalization methods: transcripts per million (TPM), normalizeGiotto (Dries, et al., 2021), SCTransform (Stuart, et al., 2019), and unit variance. We collected the normalization methods used in the current ST deconvolution methods. Since some methods require the input to be the original count matrix, we only performed the normalization benchmark on those without restrictions on the input format of ST data. Meanwhile, the normalization of the reference scRNA-seq data was kept consistent with the original requirements of the ST deconvolution methods.

Text S5 Implementation of deconvolution methods

cell2location. We followed the guidelines provided on the cell2location website: https://cell2location.readthedocs.io/en/latest/notebooks/cell2location_tutorial.html. We trained the single-cell model on the reference data with parameters max_epochs = 250 and lr = 0.002. The cell2location model was obtained on the ST data with parameters max_epochs = 30000.

DestVI. We followed the guidelines provided on the DestVI website: https://docs.scvi-tools.org/en/stable/tutorials/notebooks/DestVI_tutorial.html. For the reference data, the top 2000 high variable genes were selected. The single-cell model was trained on the reference data with parameters max_epochs = 250 and lr = 0.001. The deconvolution of ST data was implemented with parameters max_epochs = 2000.

Giotto-PAGE/rank/Hypergeometric. We followed the instructions provided on the Giotto website: https://rubd.github.io/Giotto_site/reference/runSpatialEnrich.html. The predicted results were obtained using the function 'runSpatialEnrich' with parameters enrich_mode = c("PAGE, "rank", "hypergeometric"), min_overlap_genes = 2, and top_percentage = 5.

RCTD. We followed the commands in the RCTD GitHub repository: https://github.com/dmcable/spacexr. We ran RCTD using the 'runRCTD' function in 'full mode'.

Seurat. We followed the guidelines described on the Seurat 3.2 website: https://satijalab.org/seurat/archive/v3.2/integration.html. We set normalize.method = 'SCT' in the 'FindTransferAnchors' function, and set dim = 1:30 in the 'TransferData' function.

spatialDecon. We followed the guidelines provided on the spatialDecon website: https://github.com/Nanostring-Biostats/SpatialDecon. We first created a profile matrix with reference data using the 'creat_proflie_matrix' function with the parameter minGenes = 0. The deconvolution of ST data was employed by using the 'runspatialdecon' function while setting parameter bg as 0.01.

spatialDWLS. We followed the guidelines provided on the spatialDWLS website: https://rubd.github.io/Giotto_site/articles/tut7_giotto_enrichment.html. We first clustered the spots by using the following commends createNearestNetwork (dimensions_to_use = 1:10, k = 4) and doLeidenCluster (resolution = 0.4, n_iterations = 1000). We then used the 'runDWLSDeconv' function to perform deconvolution on ST data.

SPOTlight. We followed the instructions described on the SPOTlight GitHub repository: https://marcelosua.github.io/SPOTlight/. We deconvoluted ST data using the 'spotlight_deconvolution' function with the parameters cl_n = 100, transfer = 'uv', and method = 'nsNMF'.

STdeconvolve. We followed the commands described on the STdeconvolve website: https://github.com/JEFworks-Lab/STdeconvolve. The 'restrictCorpus' function was used for selecting genes with parameters removeAbove =1.0, removeBelow = 0.05. The STdeconvolve model was fitted by the 'fitLDA' function and then the 'optimalLDA' function was used to select the optimal topic with the parameter opt = number of cell types.

stereoscope. We followed the instructions on the stereoscope GitHub repository: https://github.com/almaan/stereoscope. We first subsampled the reference data by setting the lower and upper bounds of 25 and 250 cells for each cell type, respectively. The reference model and spatial model were trained with the same parameter max_epoch = 75000.

STRIDE. We followed the commands on the STRIDE GitHub repository: https://stridespatial.readthedocs.io/en/latest/tutorials.html. The deconvolution was performed by setting the parameter '-normalize'.

Tangram. We followed the instructions described on the Tangram website: https://github.com/broadinstitute/Tangram. We set the parameters as mode = 'clusters' and density = 'rna_count_based'.

Text S6 Evaluation metrics

We calculated the following metrics to evaluate the performance of different deconvolution methods by using the known cell type proportions in the simulated datasets as the ground truth.

Assume matrices $\hat{X}_{N\times M}$ and $X_{N\times M}$ represent the predicted cell type proportions and the known cell type proportions, respectively, where N represents the number of spots and M represents the number of cell types. Accordingly, the vectors $\hat{Y}_{NM\times 1}$ and $Y_{NM\times 1}$ represent the predicted cell type proportions and the known cell type proportions. Note that $\hat{Y}_{NM\times 1} = [\hat{Y}_{N1}, \hat{Y}_{N2}, \cdots, \hat{Y}_{NM}]^T$ and $Y_{NM\times 1} = [Y_{N1}, Y_{N2}, \cdots, Y_{NM}]^T$.

(1) RMSE. The RMSE value between the predicted cell type proportion and the known cell type proportions was calculated as follows:

$$RMSE=\sqrt{\frac{1}{NM}\sum_{k=1}^{NM}(\hat{y}_k - y_k)^2}\ , \tag{S1}$$

where $\hat{y}_k$ and y_k are the k−th element of the predicted cell type proportion vector $\hat{Y}_{NM\times 1}$ and the known cell type proportions vector $Y_{NM\times 1}$, respectively. Lower RMSE value corresponds to better performance of the deconvolution method.

(2) PCC. The PCC value was calculated as follows:

$$PCC=\frac{E[(\hat{y}_k - \hat{u})(y_k - u)]}{\hat{\sigma}\sigma}, \tag{S2}$$

where $\hat{y}_k$ and y_k represent the k−th element of the predicted and known cell type proportion vectors, respectively; $\hat{u}$ and u are the mean proportions of the predicted and known cell type proportion vectors, respectively; $\hat{\sigma}$ and σ are the standard deviations of the predicted and known cell type proportion vectors, respectively. Higher PCC value corresponds to more accurate deconvolution.

(3) JSD. The JSD metric measures the similarity between two probability distributions. We calculated the JSD value of each spot as follows:

$$JSD(p_n \| q_n)=\frac{1}{2}D_{KL}\left(p_n \middle\| \frac{p_n+q_n}{2}\right)+\frac{1}{2}D_{KL}\left(q_n \middle\| \frac{p_n+q_n}{2}\right), \tag{S3}$$

$$\mathrm{D}_{KL}(a_n \| b_n)=\sum_{m=1}^{M}\left(a_{nm}\times\log\frac{a_{nm}}{b_{nm}}\right), \tag{S4}$$

where q_n and p_n represent the cell type proportions of spot n in the predicted result and the ground truth, respectively. Eq. (S4) calculates the Kullback-Leibler divergence between the two probability distributions a_n and b_n, where the predicted probability and expected probability of cell type m in spot n are represented by a_{nm} and b_{nm}, respectively. The lower JSD value indicates that the precited cell type proportions are more similar to the ground truth cell type proportions.

(4) Running time and memory. For the deconvolution methods using R language, we used the 'memory_used' function from the pryr package to evaluate the memory changes and used the 'proc.time' function to measure the running time. For the deconvolution methods using Python language, memory changes were calculated using the 'memory_full_info' function from the psutil package and the running time was assessed with the 'time' function from the time package.

(5) Score aggregation. To rank different methods, we aggregated different scores at two levels: across different datasets and different metrics. The aggregation procedure is explained in detail as follows:

For each dataset, we first normalized the values of RMSE, PCC, and JSD (50% median) by using Eq. (S5-S7), respectively.

$$score^i_{rmse} = 1 - \frac{z^i_{rmse} - \min(z^i_{rmse})}{\max(z^i_{rmse}) - \min(z^i_{rmse})}, \tag{S5}$$

$$score^i_{pcc} = \frac{z^i_{pcc} - \min(z^i_{pcc})}{\max(z^i_{pcc}) - \min(z^i_{pcc})}, \tag{S6}$$

$$score^i_{jsd} = 1 - \frac{z^i_{jsd} - \min(z^i_{jsd})}{\max(z^i_{jsd}) - \min(z^i_{jsd})}, \tag{S7}$$

where $i = 1, 2, 3$ represents the i-th synthetic dataset, z^i_{rmse}, z^i_{pcc}, and z^i_{jsd} represent the RMSE values, PCC values, and JSD (50% median) values of 14 methods on the i-th dataset, respectively. Lower RMSE and JSD correspond to higher values of $score^i_{rmse}$ and $score^i_{jsd}$, and higher PCC correspond to higher values of $score^i_{pcc}$.

After normalization, the values of each of the above scores were aggregated across different datasets by calculating their arithmetic mean, as shown in Eq. (S8-S10). The aggregated scores of different metrics were then averaged for each method by using Eq. (S11), which was used as the ultimate overall score for ranking.

$$score_{rmse} = \frac{1}{3}\sum_{i=1}^{3} score^i_{rmse}, \tag{S8}$$

$$score_{pcc} = \frac{1}{3}\sum_{i=1}^{3} score^i_{pcc}, \tag{S9}$$

$$score_{jsd} = \frac{1}{3}\sum_{i=1}^{3} score^i_{jsd}, \tag{S10}$$

$$score_{overall} = \frac{1}{3}\left(score_{rmse} + score_{pcc} + score_{jsd}\right). \tag{S11}$$

To assess the impact of variation in sequencing depths on the deconvolution accuracy of each method, we calculated the variance of the values of the above-defined $score_{overall}$ under different sequencing depths. Finally, we normalized the variance of each method by using Eq. (S12) for comparison across different methods. Lower variance

corresponds to higher $score_{variance}$ indicating that high robustness of the deconvolution method to the sequencing depth.

$$score_{variance} = 1 - \frac{var - \min(var)}{\max(var) - \min(var)}, \tag{S12}$$

In addition, the usability of each method was scored using Eq. (S13),

$$score_{usability} = \frac{1}{2}(score_{time} + score_{memory}). \tag{S13}$$

where $score_{time}$ and $score_{memory}$ for scoring time and memory are detailed in Table S1.

Figure S1

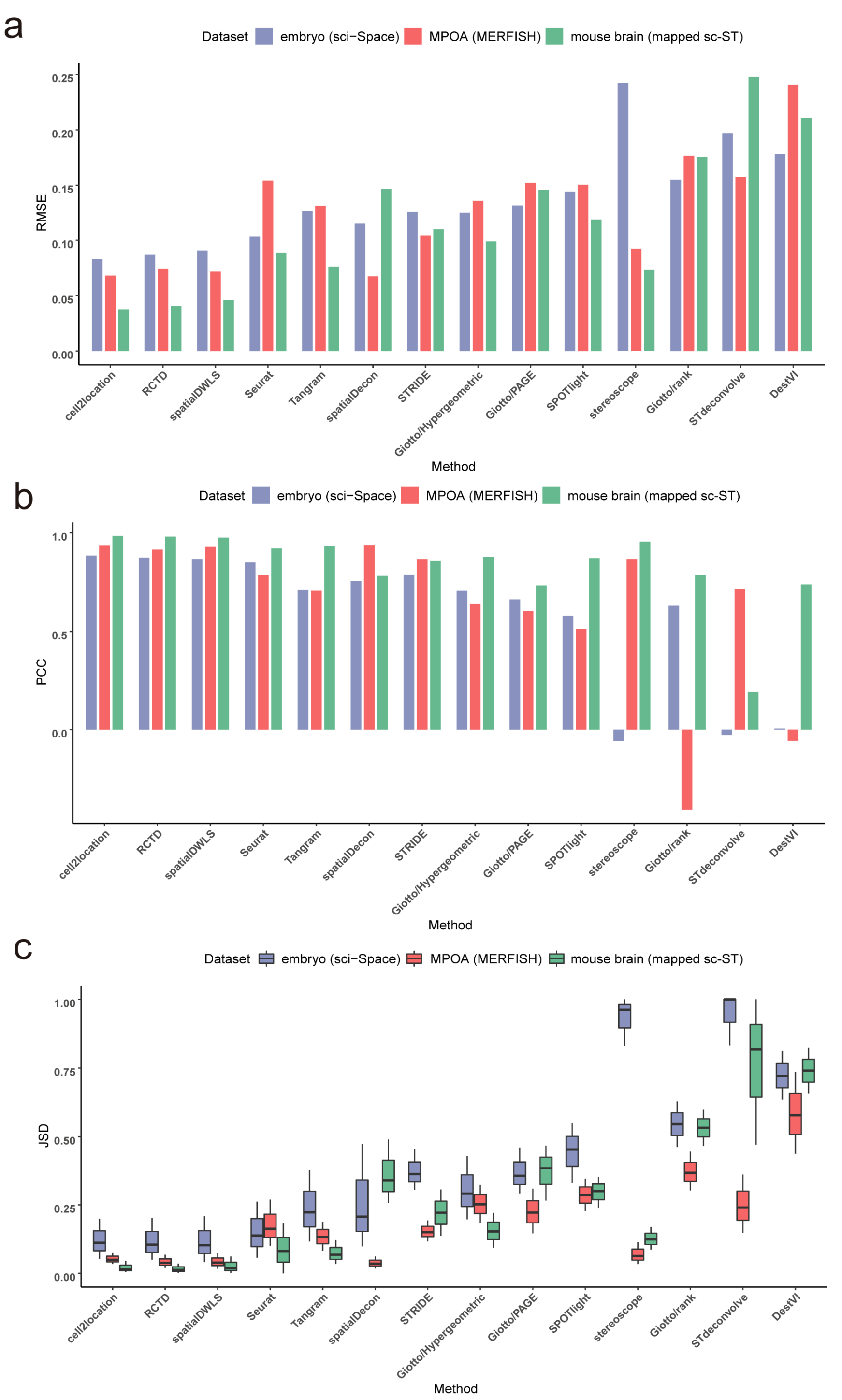

Fig. S1. Benchmarking the performance of the 14 deconvolution methods using synthetic datasets based on RMSE, PCC and JSD. RMSE (**a**), PCC (**b**), and JSD (**c**) between the ground truth proportions and the predicted proportions from different deconvolution methods were calculated. Median, lower and upper quartiles represent 50%, 25%, and 75% of JSD values, respectively.

Figure S2

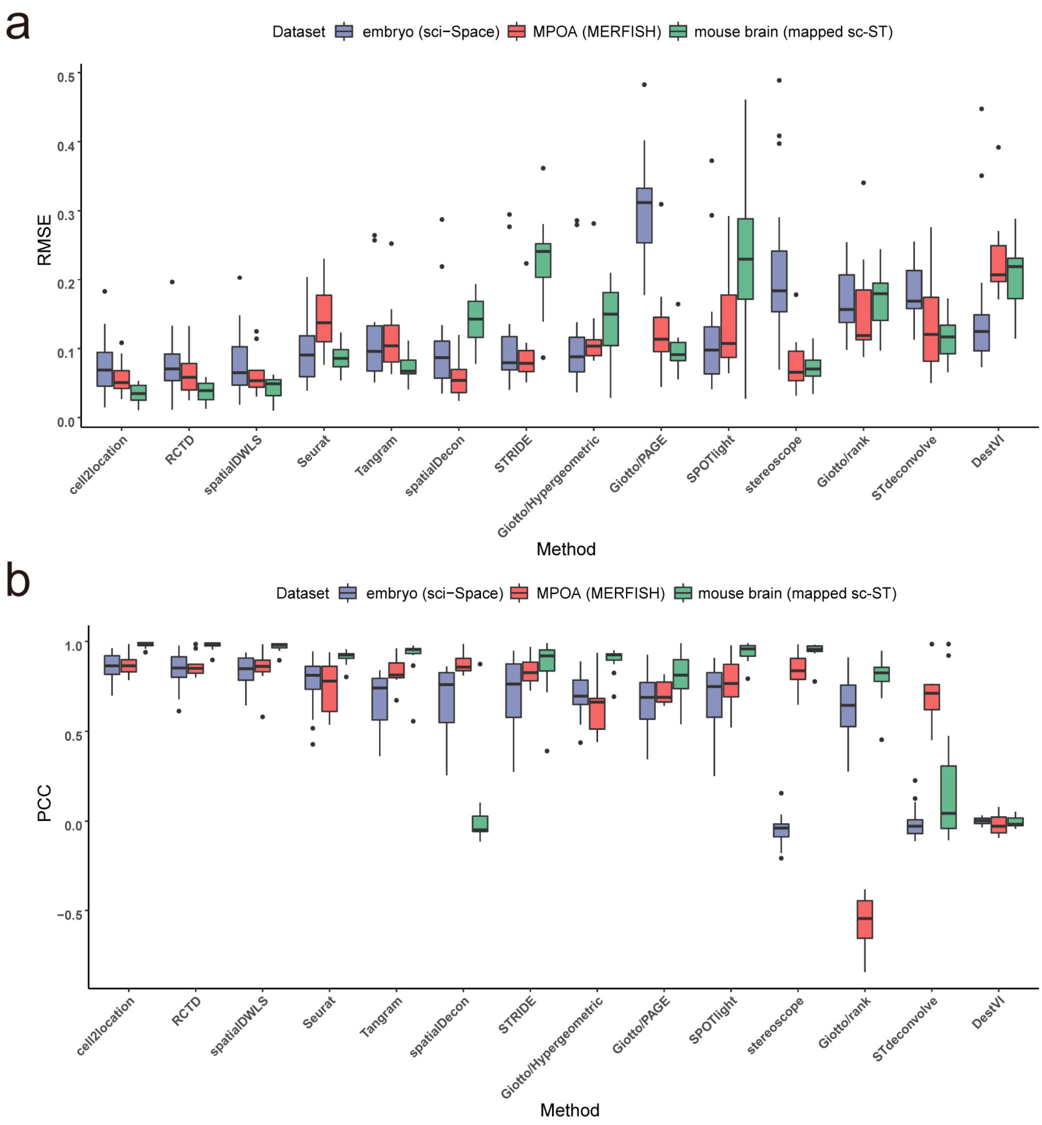

Fig. S2. Benchmarking the performance of the 14 deconvolution methods using synthetic datasets based on RMSE and PCC per cell type per cell type. RMSE per cell type (**a**), and PCC per cell type (**b**) between the ground truth proportions and the predicted proportions from different deconvolution methods were calculated.

Figure S3

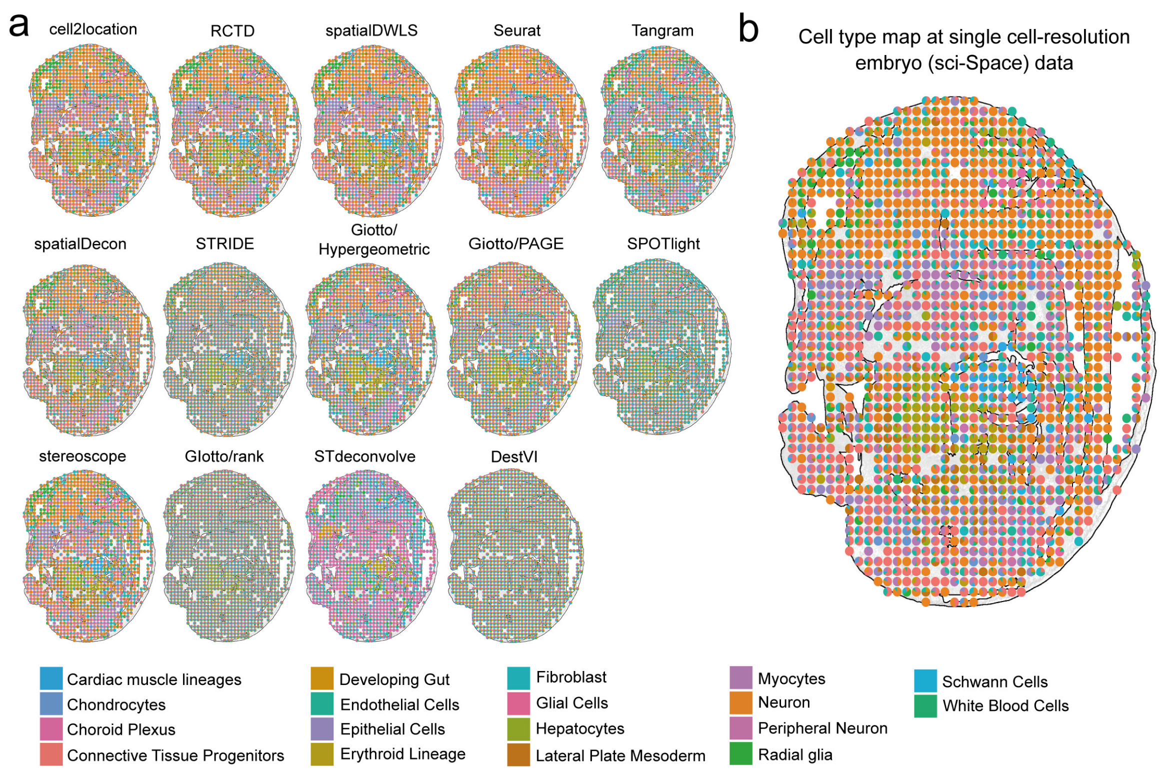

Fig. S3. Comparing the performance of ST deconvolution methods in predicting the spatial distribution of cell types on the embryo (sci-Space) dataset. (**a**) The spatial distribution of cell type proportions in the embryo (sci-Space) dataset, including the predicted results from 14 deconvolution methods. Each pie represents the simulated spot, and colors represent different cell types. (**b**) The ground truth of the cell type proportions. Since single-cell resolution embryo (sci-Space) data contains multiple cells at a capture point, each pie represents the cell type proportions at one location.

Figure S4

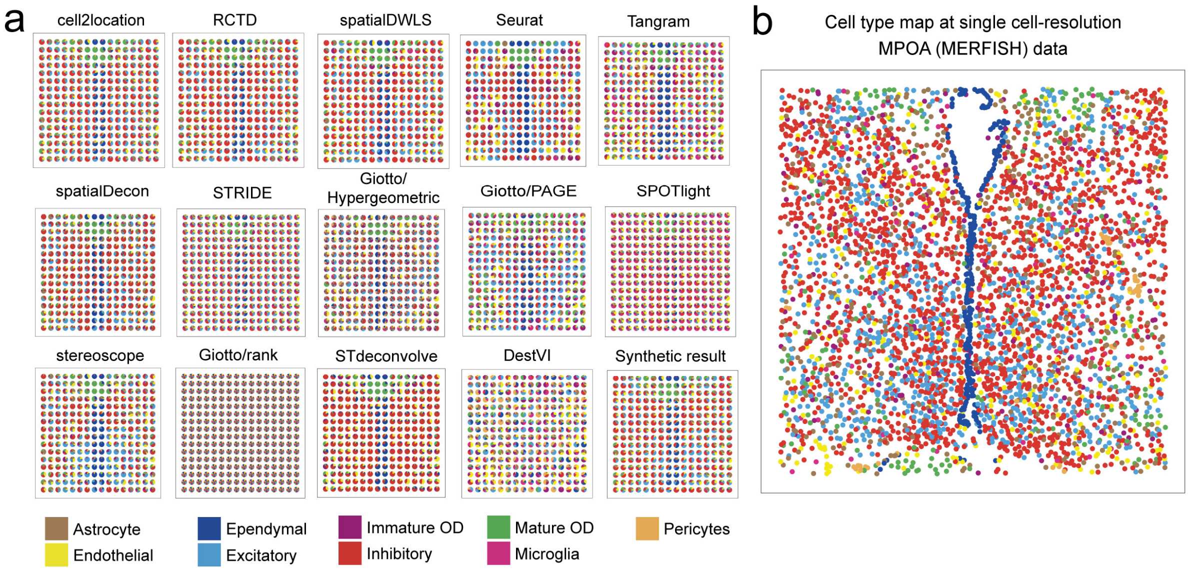

Fig. S4. Comparing the performance of ST deconvolution methods in predicting the spatial distribution of cell types on the MPOA (MERFISH) dataset. (**a**) The spatial distribution of cell type proportions from the predictions of 14 deconvolution methods and the synthetic result were represented as a spatial scatter pie chart for each simulated spot in the synthetic MPOA (MERFISH) dataset. Each pie represents the simulated spot, and colors represent different cell types. (**b**) The ground truth of cell type label at single cell-resolution for the MPOA (MERFISH) dataset.

Figure S5

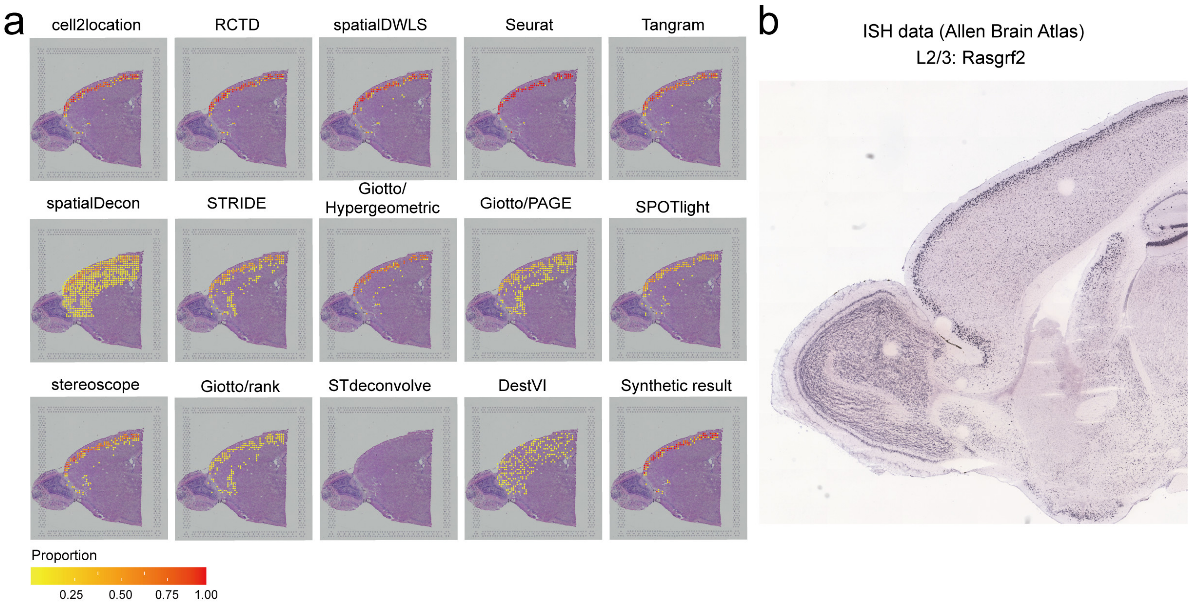

Fig. S5. Comparing the performance of ST deconvolution methods in predicting the spatial distribution of L2/3 cell type on the mouse brain (mapped sc-ST) dataset. (**a**) Proportions of the specific cortical cell type (L2/3) predicted by 14 deconvolution methods from the mouse brain (mapped sc-ST) dataset and the corresponding synthetic result. The proportion is represented by a colored dot. The redder the color, the larger the proportion. (**b**) Marker gene expression pattern in the ISH data from the Allen Brain Atlas. Rasgrf2 is a marker gene of the L2/3 cell type.

Figure S6

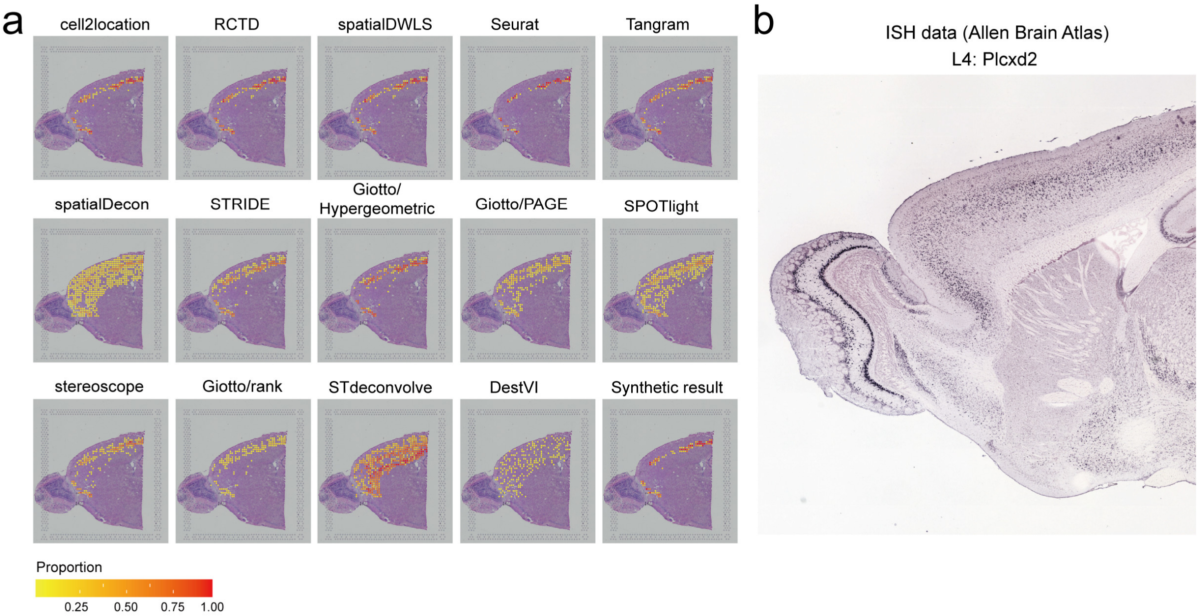

Fig. S6. Comparing the performance of ST deconvolution methods in predicting the spatial distribution of L4 cell type on the mouse brain (mapped sc-ST) dataset. (**a**) Proportions of the specific cortical cell type (L4) predicted by 14 deconvolution methods from the mouse brain (mapped sc-ST) dataset and the corresponding synthetic result. The proportion is represented by a colored dot. The redder the color, the larger the proportion. (**b**) Marker gene expression pattern in the ISH data from the Allen Brain Atlas. Plcxd2 is a marker gene of the L4 cell type.

Figure S7

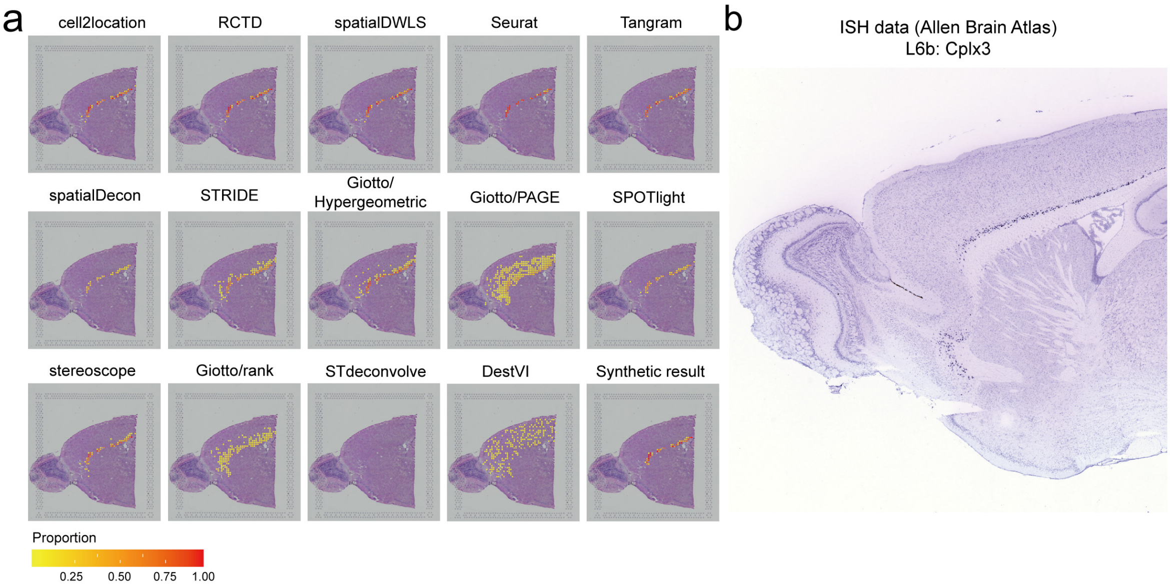

Fig. S7. Comparing the performance of ST deconvolution methods in predicting the spatial distribution of L6b cell type on the mouse brain (mapped sc-ST) dataset. (**a**) Proportions of the specific cortical cell type (L6b) predicted by 14 deconvolution methods from the mouse brain (mapped sc-ST) dataset and the corresponding synthetic result. The proportion is represented by a colored dot. The redder the color, the larger the proportion. (**b**) Marker gene expression pattern in the ISH data from the Allen Brain Atlas. Cplx3 is a marker gene of the L6b cell type.

Figure S8

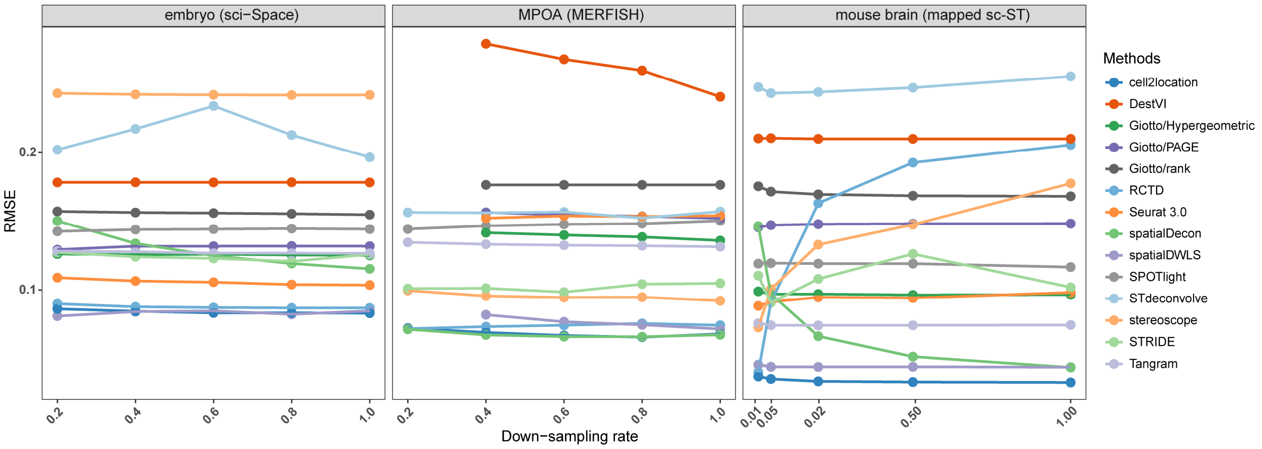

Fig. S8. Comparing the robustness of different deconvolution methods to variation of down-sampling rate. RMSE values between the expected proportion and the output proportions from different deconvolution methods were calculated for comparison. Table S2 lists details of down-sampling rates. Regarding embryo (sci-Space) and MPOA (MERFISH) datasets, cell2location, RCTD, and spatialDWLS consistently outperformed other deconvolution methods at different down-sampling rates, and most of the deconvolution methods were robust to the variation of down-sampling rates. Meanwhile, Giotto/Hypergeometric, Giotto/rank, Giotto/PAGE, and spatialDWLS could not work on the simulated dataset with the down-sampling rate = 0.2, due to the high dropout ratio of this ST dataset. For the mouse brain (mapped sc-ST) dataset, cell2location, RCTD, and spatialDWLS performed best with the down-sampling rate = 0.01, where the sequencing depth was close to that of real ST data. However, the RMSE value of RCTD gradually became larger as the down-sampling rate increased. In addition, stereoscope became gradually worse when the down-sampling rate increased, while spatialDecon became gradually worse when the down-sampling rate decreased.

Figure S9

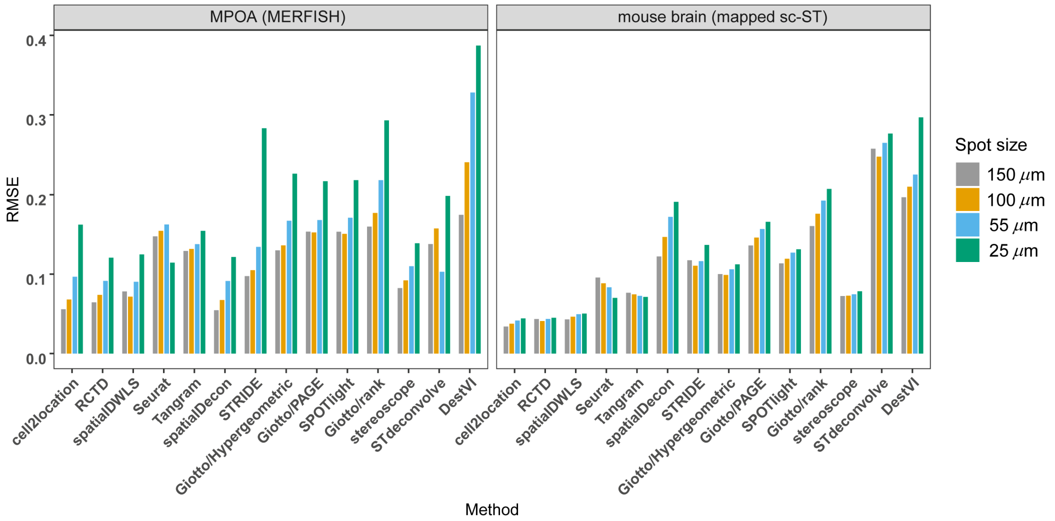

Fig. S9. Comparing the sensitivity of different deconvolution methods to different spot sizes. The bar plot represents the RMSE between the expected proportion and the output proportions from the different deconvolution methods. Different colors represent different spot sizes. Since the spots' coordinates in the embryo (sci-Space) dataset were not continuous and thus could not be used to generate synthetic datasets with different spot sizes, we adopted the MPOA (MERFISH) and mouse brain (mapped sc-ST) datasets for evaluation (see Methods). The spot with larger size contains more cells. Notably, the RMSE values of the deconvolution methods tended to become larger as the spot size decreased from 150 μm to 25 μm (Fig. 4). This may be due to that smaller size of spot contains less cells so that the sample size for inferring cell type proportions becomes even small, leading to increased bias for deconvolution.

Figure S10

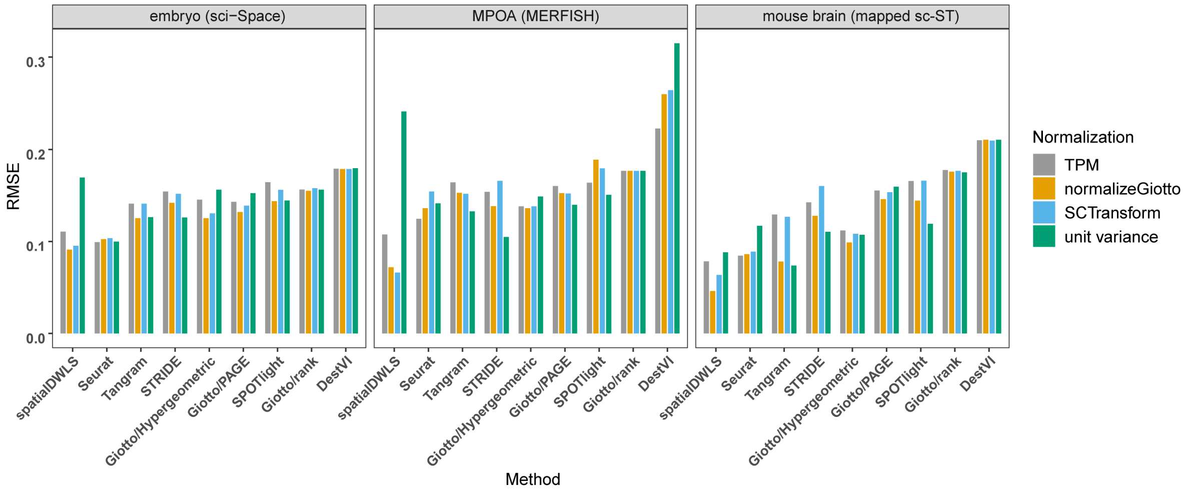

Fig. S10. Comparing the impact of different choices of ST data normalization on different deconvolution methods. RMSE values between the expected proportion and the output proportions from different deconvolution methods were calculated. Different colors represent different ST normalization methods. Because some deconvolution methods (e.g., cell2location, RCTD, spatialDecon, stereoscope, and STdeconvolve) explicitly require raw gene expression matrix as input and do not support normalization processing, we did not include these methods for investigation.

Figure S11

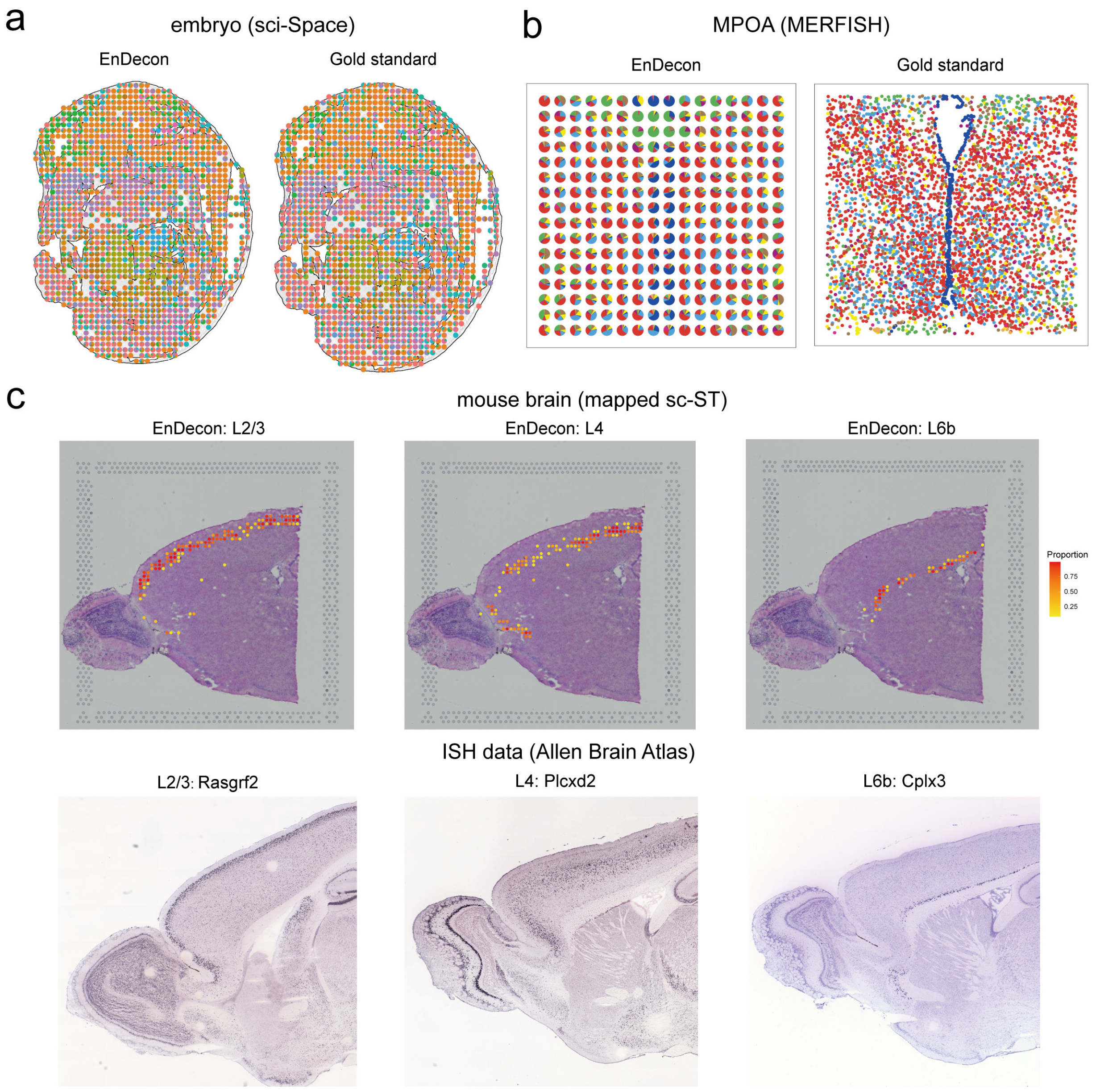

Fig. S11. Comparing the performance of the EnDecon method in predicting the spatial distribution of cell types. (**a**) The predicted proportions of EnDecon on the embryo (sci-Space) dataset compared with the gold standard from single-cell resolution embryo (sci-Space) data. Each pie represents a simulated spot colored by different cell types. The colors of cell types were consistent with Fig. S3. (**b**) The predicted proportions of EnDecon on the MPOA (MERFISH) dataset compared with the gold standard, where the gold standard was the cell type map at single-cell resolution MERFISH data. Each pie represents a simulated spot colored by different cell types. The colors of cell types were consistent with Fig. S4. (**c**) The top row shows the proportions of three cell types (i.e., L2/3, L4, L6b) estimated by EnDecon on the mouse brain (mapped sc-ST) dataset. The bottom row shows the expression patterns of the respective marker genes (i.e., Rasgrf2, Plcxd2, Cplx3) in the ISH data from the Allen Brain Atlas.

Figure S12

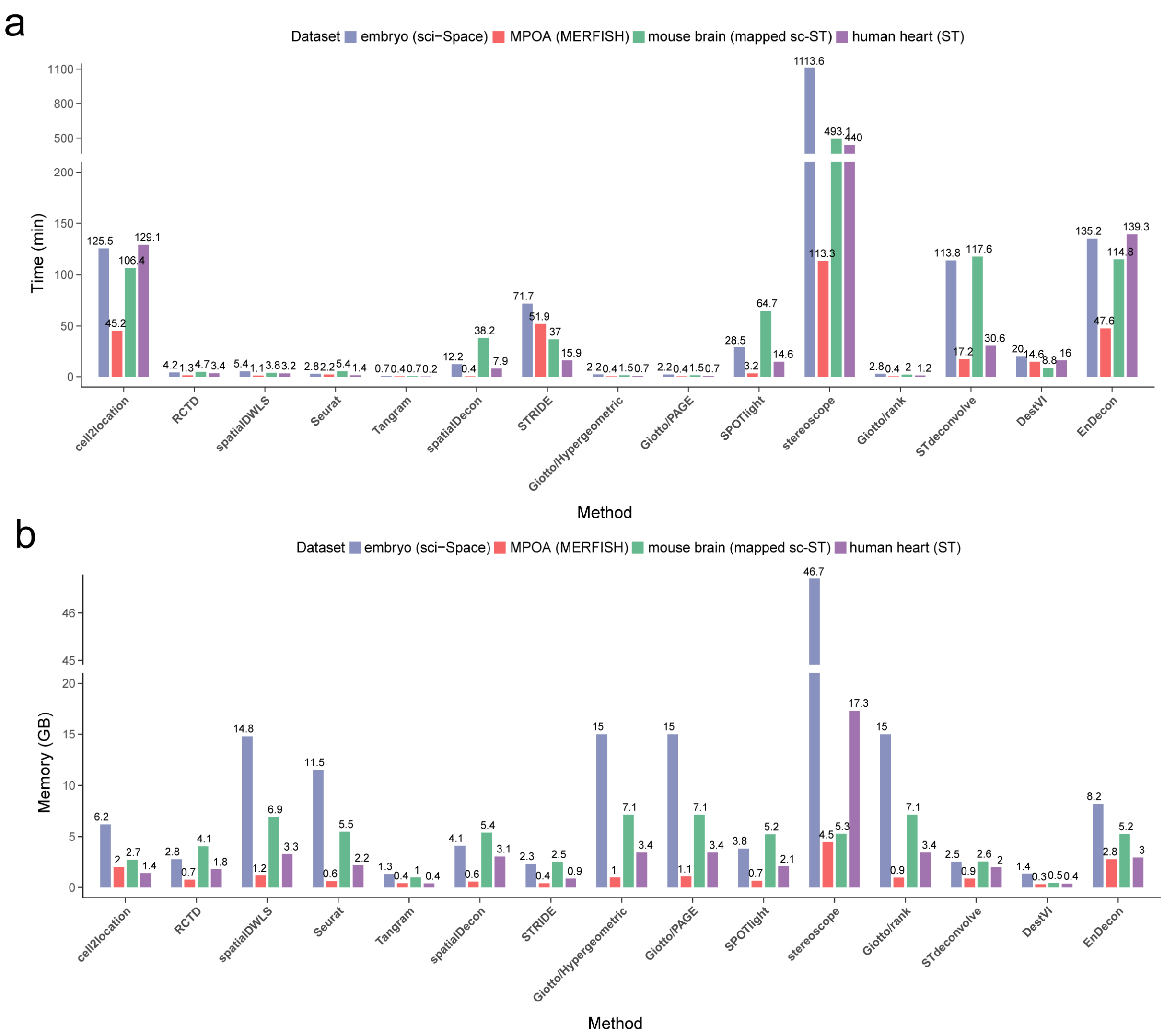

Fig. S12. Computer resources consumed by different deconvolution methods. Time requirements (min) (**a**) and Memory requirements (GB) (**b**) for different deconvolution methods across the three simulated datasets and one real ST dataset.

Table S1. Details of the criteria for calculating usability score.

Criteria for calculating usability score					
time	≤10 min	10~30 min	30~60 min	60~120 min	≥120 min
$score_{time}$	1	0.8	0.6	0.4	0.2
memory	≤1 GB	1~5 GB	5~10 GB	10~15 GB	≥15 GB
$score_{memory}$	1	0.8	0.6	0.4	0.2

Table S2. Statistics of the counts of transcripts corresponding to different down-sampling rates in the three synthetic datasets.

Dataset	Down-sampling rate	Min count	Max count	Mean count
embryo (sci-Space)	1	415	3.27e+05	4.43e+04
	0.8	332	2.62e+05	3.55e+04
	0.6	249	1.96e+05	2.66e+04
	0.4	166	1.31e+05	1.77e+04
	0.2	83	6.55e+04	8.86e+03
MPOA (MERFISH)	1	21	1.11e+04	3.94e+03
	0.8	4	8.82e+03	3.10e+03
	0.6	3	6.62e+03	2.33e+03
	0.4	1	4.42e+03	1.55e+03
	0.2	0	2.20e+03	775
mouse brain (mapped sc-ST)	1	1.00e+05	4.06e+07	1.28e+07
	0.5	5.01e+04	2.03e+07	6.38e+06
	0.2	2.00e+04	8.12e+06	2.55e+06
	0.05	5.01e+03	2.03e+06	6.38e+05
	0.01	1.00e+03	4.06e+05	1.28e+05
